# Body anthropometry affects spatiotemporal preferences in walking and running

**DOI:** 10.1242/jeb.252161

**Published:** 2026-05-19

**Authors:** Wannes Swinnen, Wouter Hoogkamer, Friedl De Groote, Benedicte Vanwanseele

**Affiliations:** ^1^ Department of Movement Sciences, KU Leuven, 3001 Leuven, Belgium; ^2^ Department of Movement and Sport Sciences, Ghent University, 9000 Ghent, Belgium; ^3^ Deparment of Kinesiology, University of Massachusetts Amherst, Amherst, MA 01003, USA

**Keywords:** Body mass, Dynamic similarity, Locomotion, Stride frequency, Ground contact time, Shoe mass

## Abstract

Despite a general similarity of walking and running gaits in healthy humans, spatiotemporal parameters vary considerably between individuals. While this variation is well recognized, the underlying causes are poorly understood. In this study, we examined whether differences in body mass, relative segment lengths (e.g. relative leg length and relative foot length) and relative segment masses (e.g. relative foot–shoe mass) contribute to the spatiotemporal variability, beyond what is accounted for by the Froude number. We collected anthropometric and spatiotemporal data from 103 trained runners (65 males, 38 females) walking (1.25 and 2 m s^−1^) and running (2–4.17 m s^−1^) on a force-measuring treadmill. Linear mixed-effects models assessed the contribution of anthropometric factors to inter-individual differences in gait. Froude number alone accounted for most of the variation in spatiotemporal variables (*R*^2^=0.71–0.92 in walking; *R*^2^=0.01–0.94 in running). Including anthropometric predictors improved model performance, particularly for variables with lower Froude dependence, increasing *R*^2^ to 0.77–0.93 (walking) and 0.16–0.94 (running). Specifically, heavier individuals and those with larger relative foot lengths exhibited longer stance times and higher duty factors (*P*≤0.033), without differences in stride frequency (*P*≥0.164). In walking, these longer stance times were primarily driven by prolonged double support time (*P*<0.001). Additionally, greater relative foot–shoe mass reduced stride frequency via longer leg swing times in both gaits (*P*≤0.007). We suggest that this spatiotemporal variability reflects individual strategies to minimize the metabolic cost of locomotion by adjusting the trade-off between stance-phase and swing-phase metabolic demands.

## Teaser

**Summary:** Inter-individual differences in body mass, relative foot length and relative foot–shoe mass contribute to variability in humans' preferred spatiotemporal patterns during walking and running.

## INTRODUCTION

Humans walk and run in a fundamentally similar way – placing one foot in front of the other to move forward. Yet, despite this shared general locomotor pattern, individuals exhibit substantial variability in how they put one foot in front of the other. This variability is often recognizable, allowing us to identify friends or relatives by their gait (Loula et al., 2005). Specifically, spatiotemporal gait characteristics such as step frequency can vary by more than 20% between individuals walking or running at matched speeds (Cavanagh and Kram, 1989; Swinnen et al., 2021; van der Zee et al., 2022). Remarkably, while these inter-individual differences are well recognized, the underlying causes of spatiotemporal gait variability remain poorly understood.

Seminal work from Alexander and Jayes (1983) introduced dynamic similarity theory to explain differences in gait characteristic between animals when moving at the same (absolute) speed. Dynamic similarity theory postulates that animals moving at a dynamically similar speed, e.g. matched Froude number, would exhibit a similar gait pattern and are therefore expected to have equal spatiotemporal gait characteristics when these are rendered dimensionless (Alexander and Jayes, 1983). Yet, a general assumption for dynamic similarity is that these animals are geometrically similar such that linear dimensions scale with body mass^1/3^ and all relative segment lengths and masses are constant. Consequently, while dynamic similarity theory may account for differences in body size through the dependence of Froude number on leg length, systematic deviations from dynamic similarity are expected when individuals deviate from geometric similarity; for example, through variation in relative segment lengths or relative segment masses (e.g. mass distributions). Indeed, when bipedal birds run at dynamically similar speeds, larger (e.g. heavier) birds use relatively shorter strides and higher stride frequencies than smaller birds. Conversely, birds with long legs relative to their mass tend to adopt longer strides as a result of longer leg swing times (Daley and Birn-Jeffery, 2018).

Humans vary widely in body shape and size, and anthropometry is therefore often proposed as a key determinant of habitual gait patterns (Reenalda et al., 2016; van der Walt and Wyndham, 1973). Yet, previous studies yielded inconsistent associations between inter-individual differences in basic anthropometric measures such as body height or leg length and stride frequency, with reported correlations ranging from strongly negative (*r*=−0.77) to negligible (*r*=0.1) in relatively small sample sizes (Cavanagh and Kram, 1989; Cavanagh and Williams, 1982; Cavanagh et al., 1977; Elliott and Blanksby, 1979; Roche-Seruendo et al., 2019; van der Walt and Wyndham, 1973). These inconsistencies may reflect limitations of using overall measures of body length, rather than also considering relative segment lengths and mass distributions. From a scaling perspective, humans can be expected to be geometrically dissimilar, with relative segment lengths and segment masses varying substantially across individuals. Therefore, dimensionless gait characteristics may show residual inter-individual variability, after accounting for Froude number, when these assumptions of geometric similarity are violated.

In contrast to the rather limited insights into the factors contributing to spatiotemporal differences between individuals, there is ample evidence that humans tend to adopt a metabolically optimal gait pattern (Arellano and Kram, 2011; Cavanagh and Williams, 1982; Donelan et al., 2001; Selinger et al., 2015; Swinnen et al., 2022; Umberger and Martin, 2007), and that anthropometry can explain a substantial proportion of the variation in running economy (Black et al., 2020). It is well established that preferred stride frequency closely corresponds with optimal frequency in walking (Minetti et al., 1995; Umberger and Martin, 2007; Zarrugh and Radcliffe, 1978) and running (Cavanagh and Williams, 1982; Högberg, 1952; Swinnen et al., 2021), and that deviations from one's habitual duty factor (the proportion of stride time spent in stance) incur a metabolic penalty (Bonnaerens et al., 2019). The metabolic cost of walking is largely determined by step-to-step transition cost, i.e. the metabolic cost to redirect the body's center of mass (COM) velocity from one pendulum arc to the next (Donelan et al., 2002; Gottschall and Kram, 2005; Kuo et al., 2005), whereas the metabolic cost of running is dominated by body weight support and propulsion (Arellano and Kram, 2014; Chang and Kram, 1999; Teunissen et al., 2007). Consequently, for both gaits, the stance phase is metabolically the most expensive phase whereas leg swing has a smaller metabolic cost, with previous studies estimating the cost of leg swing to account for ∼10–20% of the metabolic cost in walking (Gottschall and Kram, 2005; Griffin et al., 2003) and 7–26% in running (Arellano and Kram, 2014; Marsh et al., 2004; Modica and Kram, 2005). However, some anthropometric characteristics may disproportionately affect the metabolic cost of either stance or swing, thereby potentially shifting the optimal and preferred locomotor pattern and contributing to inter-individual variability in spatiotemporal gait parameters.

Studies on the effects of external load on locomotion further highlight body mass and relative foot mass as potentially important determinants of spatiotemporal gait variability. These studies illustrate that the metabolic cost of carrying additional mass strongly depends on its location on the body (Browning et al., 2007; Coifman et al., 2024a; Martin, 1985; Schertzer and Riemer, 2014). Adding mass distally (e.g. around the feet) is metabolically much more expensive than adding the same mass near the body's COM (Browning et al., 2007; Coifman et al., 2024a; Martin, 1985; Schertzer and Riemer, 2014), emphasizing that the cost of leg swing can substantially contribute to whole-body metabolic cost during locomotion. This phenomenon reflects the inertial cost of swinging a limb: distal mass increases the limb's moment of inertia, requiring greater muscular effort to accelerate and decelerate the leg during swing. Comparative studies show that cursorial mammals have relatively reduced distal limb mass compared with non-cursorial species, minimizing limb moment of inertia and swing cost (Kilbourne and Hoffman, 2015). Additionally, some of these load-carrying studies report the effect of added mass on spatiotemporal variables. Adding mass at the waist does not affect stride frequency in walking or running, yet it increases the adopted duty factor (Browning et al., 2007; Griffin et al., 2003; Werkhausen et al., 2019). In contrast, for every kilogram added to each foot, step frequency reduces by 2.5–3% when walking (Browning et al., 2007; Holt et al., 1990) and by 2–2.5% when running (Coifman et al., 2024b; Martin, 1985). Interestingly, these reductions in step frequency are mainly caused by increases in leg swing time, with minimal changes in stance time (Browning et al., 2007; Coifman et al., 2024b; Martin, 1985). Furthermore, Reenalda et al. (2016) demonstrated that the optimal stride frequency in running shifts to ∼5% slower frequencies when a 1 kg mass is added to each foot compared with when the same mass is attached at the waist. Although these studies examined the acute effects of added mass, they collectively suggest that body mass and relative foot mass determine spatiotemporal gait variability beyond what would be expected from dynamic similarity theory.

In this study, we examined the extent to which anthropometric characteristics explain inter-individual variability in spatiotemporal gait parameters (e.g. stride frequency, stride length, ground contact time, leg swing time, duty factor and double support time) across walking and running speeds. While dynamic similarity theory provides a foundational framework for predicting gait patterns based on geometric and kinematic scaling (e.g. via Froude number), substantial variability persists even among individuals moving at dynamically similar speeds. We therefore tested whether anthropometric differences account for the residual variability in dimensionless spatiotemporal variables after controlling for Froude number, thereby evaluating how deviations from geometric similarity contribute to violations of dynamic similarity predictions. Specifically, we assessed the influence of body mass, relative segment length (e.g. relative leg length and relative foot length), relative segment mass (e.g. relative foot–shoe mass) and sex. We hypothesized that, although Froude number (e.g. locomotion speed) is a primary determinant of an individual's spatiotemporal gait preference across different speeds, greater body mass would be associated with increased stance time and a higher duty factor. Additionally, we expected that greater relative foot–shoe mass would reduce stride frequency by prolonging leg swing time, consequently reducing duty factor. By modeling these relationships within a geometric and dynamic similarity theory framework, we aimed to clarify how anthropometric variation from idealized scaling relationships contributes to inter-individual differences in gait.

## MATERIALS AND METHODS

### Experimental design

We collected anthropometric and spatiotemporal data of 103 runners (65 males, 38 females; Table 1) aged between 18 and 56 years, who ran at least 16 km per week on average, had not actively tried to adapt their locomotion pattern (e.g. engaged in cadence training) and were injury free at the time of testing. All subjects participated voluntarily and gave written informed consent, approved by the KU Leuven social and societal ethics committee (G-2022-5673), prior to experimental data collection. The experimental data collection consisted of two parts: first we recorded anthropometric data and next we collected spatiotemporal data at different locomotion speeds. After a short warm-up (≥5 min) and when participants felt comfortable while walking and running on the force-measuring split-belt treadmill (M-gait, Motekforce Link, Houten, The Netherlands), we collected data for typical walking (1.25 m s^−1^), fast walking (2.00 m s^−1^) and running at a range of speeds (2.00, 2.50, 3.33 and 4.17 m s^−1^). Walking trials were performed while walking with each foot on a separate belt whereas during the running trials, participants were asked to run on only one belt. In cases where participants used a (near) grounded running style (e.g. no flight phase), they were asked to run with each foot on a separate belt to ensure that reliable spatiotemporal data could be collected. During experimental testing, participants wore standardized running shoes (Li Ning marathon, shoe mass: 176–264.5 g per shoe depending on size), except for one participant who did not fit into the provided standard running shoes and therefore ran with her own running shoes (shoe mass=217.5 g per shoe). Additionally, one participant did not feel comfortable running at 4.17 m s^−1^ and this speed was excluded for that specific individual.

**Table 1.** Subject demographics

	Mean±s.d. (range)
Body mass (kg)	68.6±9.2 (47.2–90.6)
Body height (cm)	176.8±8.1 (156.9–193.0)
BMI	21.9±1.9 (17.5–26.7)
Leg length (m)	0.91±0.04 (0.795–1.03)
Relative leg length	0.515±0.011 (0.492–0.543)
Relative foot–shoe mass	0.0163±0.0013 (0.0133–0.0195)
Relative foot length	0.279±0.013 (0.250–0.307)
Age (years)	31.9±8.9 (18–56)
Running experience (years)	13.9±7.4 (1–30)
Average weekly distance (km per week)	42.9±21.4 (16–110)

BMI, body mass index.

### Anthropometry

We measured five different anthropometric variables: height, leg length (trochanter to ground), foot length, body mass and foot volume. Foot volume was determined by submersion of the foot up to the lateral malleolus into a specifically designed water-filled bucket (bucket dimensions: 120×300×250 mm; Atelier Wauters, Marcinelle, Belgium). The rise of the water level was measured and converted into a volume (coefficient of variation<3%). The volume of both feet was measured bilaterally and the average across both sides was used as foot volume. Next, foot volume was converted to mass by multiplying the volume by 1.1 kg l^−1^ (Visser et al., 1997). Estimated foot and shoe mass were summed to acquire the corresponding foot–shoe mass and divided by body mass to acquire relative foot–shoe mass. Furthermore, we computed relative leg length by dividing leg length by height, and relative foot length by dividing foot length by leg length.

### Spatiotemporal locomotion characteristics

We used ground reaction force data, sampled at 1000 Hz, to determine individual spatiotemporal gait variables. For walking trials, ground reaction force data were low-pass filtered at 6 Hz and a force threshold of 30 N was adopted to determine stance. For running trials, ground reaction force data were low-pass filtered at 20 Hz and a 50 N threshold was adopted to determine stance. Stride time was defined as the time between two consecutive initial foot contacts of the same foot. Subsequently, stride length (stride time×speed), stride frequency (1/stride time) and duty factor (ground contact time/stride time) were calculated. All spatiotemporal variables were collected for at least 36 steps (122 steps on average) and we considered the average of each spatiotemporal variable as the individual's preferred spatiotemporal variable.

### Statistics

To investigate how individual anthropometric characteristics influence spatiotemporal gait variables beyond predictions from dynamic similarity theory, we used linear mixed-effects models. First, gait speed (*v*) was converted into Froude number (*Fr*) using leg length (*L*) and gravitational acceleration (***g***):
(1)

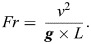
Next, all spatiotemporal variables were converted into dimensionless quantities using gravitational acceleration and leg length:
(2)

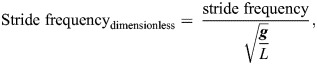

(3)

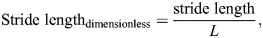

(4)

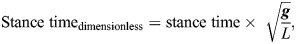

(5)

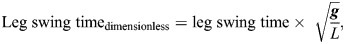

(6)

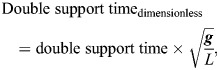
where, in the calculations, stride frequency was measured in Hz, stride length in m, stance time in s, leg swing time in s and double support time in s. Duty factor is inherently dimensionless and was therefore not transformed.

For each dimensionless spatiotemporal variable and each locomotion mode, we fitted two linear mixed-effects models. In the first model, we only included Froude number, computed individually for each participant and speed, as the sole fixed effect. This model quantifies how much of the spatiotemporal variability is explained by Froude number across all walking and running speeds: 

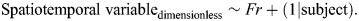


In the second model, we also included individuals' anthropometric characteristics and sex as well as the interaction effects between Froude number and these anthropometric characteristics:

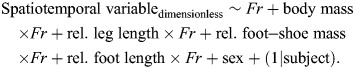
In this second model, significantly non-zero coefficients for each of these terms indicate a significant influence on the corresponding spatiotemporal variable. We assessed collinearity among anthropometric predictors using a principal component analysis. All four anthropometric characteristics contributed substantially to the inter-individual variability, justifying their inclusion (Fig. S1). Initially, interaction terms between anthropometric characteristics and Froude number were included to test for whether anthropometric effects were dependent on Froude number (i.e. speed). However, non-significant interactions were removed to simplify the model, retaining only main effects. To quantify the proportion of variance accounted for by the fixed effects, we computed the marginal *R*^2^. To support interpretation of the mixed-effects results, we additionally show Froude-adjusted regression plots that visualize the relationship between each anthropometric variable and the Froude-adjusted spatiotemporal outcome. These plots remove only the fixed effect of Froude number and therefore do not account for shared variance among anthropometric predictors or for potential interactions. As a result, their visual trends may differ from the effects estimated in the mixed-effects models (model 2). To more directly illustrate the effects estimated by our statistical approach, we also provide added-variable (partial regression) plots in Figs S2 and S3. These plots show the unique contribution of each anthropometric predictor after removing the fixed effect of Froude number and adjusting for the other anthropometric variables in the model. Statistical significance was set at *P*<0.05. The full dataset is provided (Dataset 1).

## RESULTS

**Fig. 1.**
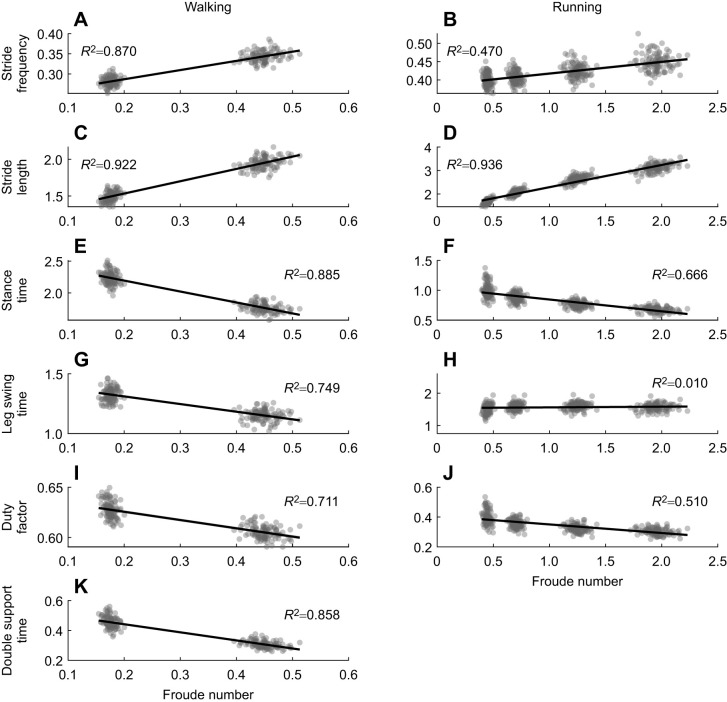
**Dimensionless spatiotemporal gait variables as a function of Froude number during walking (left) and running (right).** (A,B) Dimensionless stride frequency; (C,D) dimensionless stride length; (E,F) dimensionless stance time; (G,H) dimensionless leg swing time; (I,J) duty factor; and (K) dimensionless double support time. Gray dots represent individual observations (*n*=103) at two walking speeds and four running speeds (*n*=102 at the highest running speed). Black lines show the fixed-effects predictions from the first linear mixed-effect model: spatiotemporal variable_dimensionless_∼*Fr*+(1|subject); and shaded bands (barely visible because of their small width) show the 95% confidence intervals of the mean predicted value. Marginal *R*^2^ values quantify the proportion of variance explained by Froude number.

**Fig. 2.**
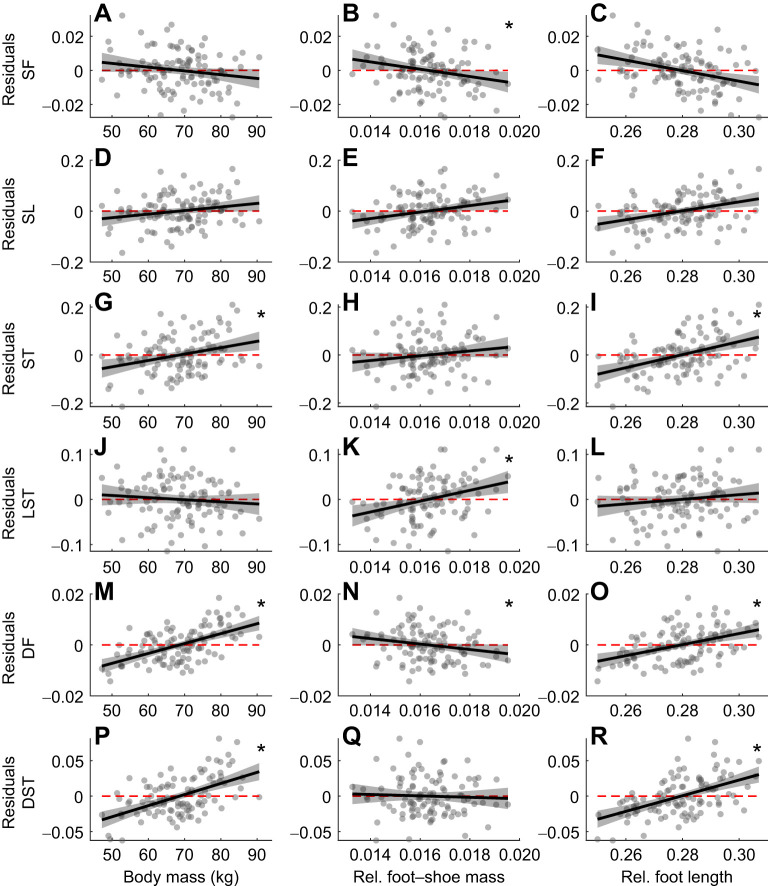
**Relationship between Froude-adjusted residuals of dimensionless spatiotemporal gait variables and individual anthropometric measures during walking.** Residuals for dimensionless stride frequency (SF; A–C), dimensionless stride length (SL; D–F), dimensionless stance time (ST; G–I), dimensionless leg swing time (LST; J–L), duty factor (DF; M–O) and dimensionless double support time (DST; P–R) represent the component of each variable not explained by Froude number, plotted against body mass (left), relative foot–shoe mass (center) and relative foot length (right). Each point shows the subject–level mean residual across walking speeds (*n*=103). Black lines show simple linear regressions with 95% confidence intervals of the mean predicted value. Asterisks denote anthropometric predictors that showed significant main effects in the mixed-effects model (Table 3). Note that these Froude-adjusted univariate relationships may differ from the statistical results reported in Table 3, as they do not account for shared variance among anthropometric variables or potential interaction effects. For clarity, relative leg length is not shown because it did not exhibit a significant main effect in the linear mixed-effects model.

**Fig. 3.**
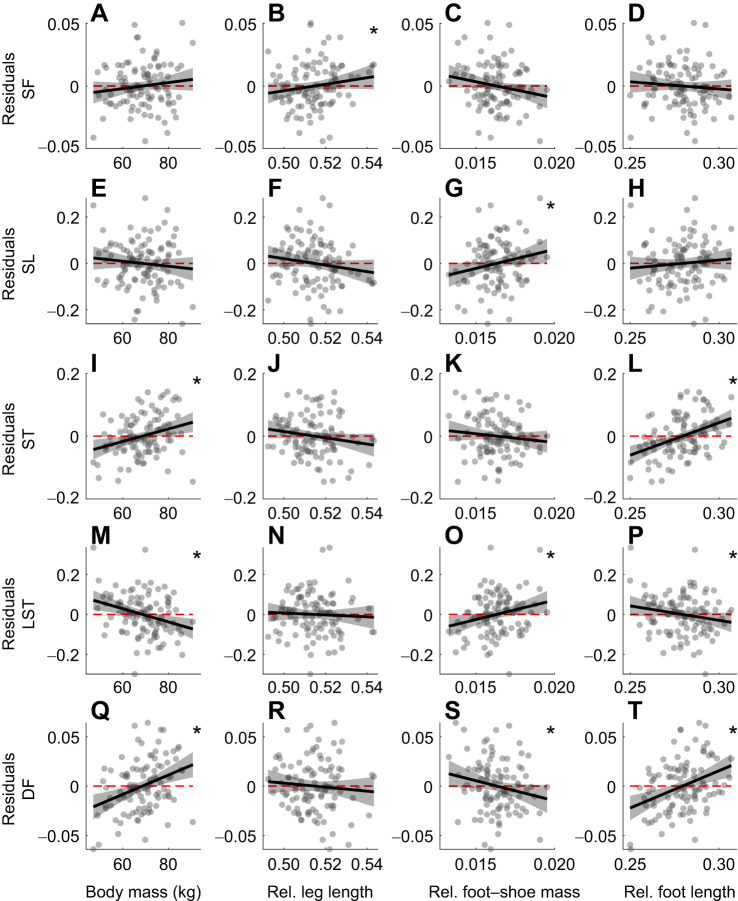
**Relationship between Froude-adjusted residuals of dimensionless spatiotemporal gait variables and individual anthropometric measures during running.** Residuals for dimensionless stride frequency (SF; A–D), dimensionless stride length (SL; E–H), dimensionless stance time (ST; I–L), dimensionless leg swing time (LST; M–P) and duty factor (DF; Q–T) represent the component of each variable not explained by Froude number, plotted against body mass (left), relative leg length (center left), relative foot–shoe mass (center right) and relative foot length (right). Each point shows the subject–level mean residual across running speeds (*n*=103). Black lines show simple linear regressions with 95% confidence intervals of the mean predicted value. Asterisks denote anthropometric predictors that showed significant main effects in the mixed-effects model (Table 4). Note that these Froude-adjusted univariate relationships may differ from the statistical results reported in Table 4, as they do not account for shared variance among anthropometric variables or potential interaction effects.

Spatiotemporal gait data for walking and running at each speed are presented in Table 2. As expected, when testing the linear mixed effect models that included only Froude number as a predictor, its coefficient was always significantly different from zero (*P*<0.001). The proportion of spatiotemporal variance accounted for by Froude number alone ranged from *R*^2^=0.711 to 0.922 in walking and *R*^2^=0.010 to 0.936 in running (Fig. 1). In the next paragraphs, we describe which anthropometric characteristics significantly contributed to explaining spatiotemporal gait variability beyond what Froude number could capture, using the second linear mixed-effect model (Tables 3 and 4 and Figs 2 and 3). To illustrate the influence of each significant anthropometric factor on the spatiotemporal gait preferences, we used an ‘average’ individual (all anthropometric values equal to the sample mean) as a reference and simulated how an isolated 10% increase in each characteristic affected the spatiotemporal gait preferences (Tables S1 and S2).

**Table 2.** Spatiotemporal variables and corresponding Froude numbers while walking and running at different speeds

	Walking		Running			
	1.25 m s^−1^	2 m s^−1^	2 m s^−1^	2.5 m s^−1^	3.33 m s^−1^	4.17 m s^−1^
Froude no.	0.175±0.01	0.45±0.02	0.45±0.02	0.70±0.03	1.25±0.06	1.95±0.09
Stride frequency (Hz)	0.924±0.04 (0.82–1.04)	1.13±0.06 (1.01–1.28)	1.32±0.06 (1.19–1.45)	1.34±0.06 (1.20–1.47)	1.39±0.07 (1.21–1.55)	1.47±0.08 (1.27–1.67)
Stride length (m)	1.36±0.07 (1.20–1.53)	1.77±0.09 (1.56–1.98)	1.52±0.07 (1.38–1.68)	1.87±0.08 (1.70–2.09)	2.39±0.12 (2.14–2.75)	2.84±0.16 (2.50–3.28)
Stance time (ms)	681±37 (587–775)	537±28 (473–600)	302±37 (223–429)	267±22 (215–316)	233±17 (196–276)	206±15 (170–247)
Leg swing time (ms)	404±18 (354–451)	350±17 (307–391)	459±40 (353–561)	481±31 (399–579)	486±33 (398–602)	475±34 (398–589)
Duty factor	0.628±0.008 (0.611–0.650)	0.605±0.007 (0.591–0.621)	0.397±0.045 (0.295–0.535)	0.358±0.027 (0.283–0.417)	0.324±0.024 (0.265–0.382)	0.303±0.022 (0.252–0.353)
Double support time (ms)	139±13 (116–171)	93±8 (108–171)	0.3±2.8 (0–28)	–	–	–

Data are means±s.d. with range in parentheses.

**Table 3.** Coefficients and corresponding *P*-values for the effect of body mass, relative leg length, relative foot mass, relative foot length and sex on dimensionless spatiotemporal walking preferences

Variable	Factor	Estimate	*P*-value	*R* ^2^
Stride frequency	Intercept	2.21×10^−1^	**0.003**	0.885
	Froude no.	2.28×10^−1^	**<0.001**	
	Body mass	−2.61×10^−4^	0.164	
	Rel. leg length	2.26×10^−1^	0.061	
	Rel. foot mass	−2.51	**0.031**	
	Rel. foot length	−1.44×10^−1^	0.222	
	Sex (Male)	5.28×10^−3^	0.149	
Stride length	Intercept	1.53	**<0.001**	0.932
	Froude no.	7.04×10^−1^	**0.008**	
	Body mass	5.52×10^−4^	0.638	
	Rel. leg length	−1.10	0.096	
	Rel. foot mass	3.92	0.609	
	Rel. foot length	7.84×10^−1^	0.225	
	Sex (Male)	−2.50×10^−2^	0.211	
	Froude no.×body mass	6.63×10^−3^	**<0.001**	
	Froude no.×rel. foot mass	32.3	**0.022**	
Stance time	Intercept	2.18	**<0.001**	0.909
	Froude no.	−9.85×10^−1^	**<0.001**	
	Body mass	6.20×10^−3^	**<0.001**	
	Rel. leg length	−1.36	0.092	
	Rel. foot mass	11.8	0.128	
	Rel. foot length	1.69	**0.033**	
	Sex (Male)	−4.56×10^−2^	0.063	
	Froude no.×body mass	−1.08×10^−2^	**<0.001**	
Leg swing time	Intercept	1.73	**<0.001**	0.770
	Froude no.	−6.40×10^−1^	**<0.001**	
	Body mass	−2.87×10^−4^	0.716	
	Rel. leg length	−9.44×10^−1^	0.064	
	Rel. foot mass	13.6	**0.006**	
	Rel. foot length	−9.06×10^−2^	0.985	
	Sex (Male)	−1.01×10^−2^	0.513	
Duty factor	Intercept	4.82×10^−1^	**<0.001**	0.826
	Froude no.	2.19×10^−1^	**0.026**	
	Body mass	7.63×10^−4^	**<0.001**	
	Rel. leg length	1.45×10^−1^	0.071	
	Rel. foot mass	−1.20	**0.030**	
	Rel. foot length	1.97×10^−1^	**<0.001**	
	Sex (Male)	−3.13×10^−3^	0.073	
	Froude no.×body mass	−1.28×10^−3^	**<0.001**	
	Froude no.×rel. leg length	−4.15×10^−1^	**0.024**	
Double support time	Intercept	−9.31×10^−2^	0.617	0.916
	Froude no.	8.43×10^−1^	**0.025**	
	Body mass	3.67×10^−3^	**<0.001**	
	Rel. leg length	3.48×10^−1^	0.282	
	Rel. foot mass	−8.86×10^−1^	0.703	
	Rel. foot length	8.53×10^−1^	**<0.001**	
	Sex (Male)	−1.78×10^−2^	**0.017**	
	Froude no.×body mass	−6.82×10^−3^	**<0.001**	
	Froude no.×rel. leg length	−1.79	**0.011**	

Interaction effects between Froude number and anthropometric measure were only included in the model when significant. Marginal *R*^2^ represents the variance accounted for by the fixed effects of the linear mixed-effect model. Bold indicates significance.

**Table 4.** Coefficients and corresponding *P*-value for the effect of body mass, relative leg length, relative foot mass, relative foot length and sex on dimensionless spatiotemporal running preferences

Variable	Factor	Estimate	*P*-value	*R* ^2^
Stride frequency	Intercept	1.81×10^−1^	0.129	0.505
	Froude no.	8.34×10^−2^	**<0.001**	
	Body mass	1.72×10^−5^	0.955	
	Rel. leg length	4.05×10^−1^	**0.039**	
	Rel. foot mass	−5.96×10^−1^	0.766	
	Rel. foot length	−4.44×10^−3^	0.982	
	Sex (Male)	9.05×10^−3^	0.129	
	Froude no.×rel. foot mass	−3.15	**<0.001**	
Stride length	Intercept	2.82	**<0.001**	0.939
	Froude no.	3.02×10^−1^	**0.045**	
	Body mass	−2.30×10^−3^	0.267	
	Rel. leg length	−2.14	0.056	
	Rel. foot mass	−9.50	0.489	
	Rel. foot length	−1.15×10^−1^	0.916	
	Sex (Male)	−4.27×10^−2^	0.207	
	Froude no.×body mass	2.11×10^−3^	**0.049**	
	Froude no.×rel. foot mass	30.6	**<0.001**	
Stance time	Intercept	3.55×10^−2^	0.928	0.735
	Froude no.	1.94×10^−1^	0.063	
	Body mass	4.16×10^−3^	**<0.001**	
	Rel. leg length	−2.32×10^−1^	0.710	
	Rel. foot mass	−5.31	0.377	
	Rel. foot length	3.44	**<0.001**	
	Sex (Male)	−5.36×10^−2^	**0.006**	
	Froude no.×body mass	−1.84×10^−3^	**0.002**	
	Froude no.×rel. foot length	−9.53×10^−1^	**0.025**	
Leg swing time	Intercept	3.17	**<0.001**	0.160
	Froude no.	−1.58×10^−1^	**<0.001**	
	Body mass	−4.75×10^−3^	**0.010**	
	Rel. leg length	−2.11	0.052	
	Rel. foot mass	28.5	**0.007**	
	Rel. foot length	−2.44	**0.023**	
	Sex (Male)	−4.52×10^−3^	0.890	
	Froude no.×body mass	2.63×10^−2^	**<0.001**	
Duty factor	Intercept	−1.20×10^−1^	0.459	0.615
	Froude no.	9.94×10^−2^	**0.015**	
	Body mass	1.56×10^−3^	**0.001**	
	Rel. leg length	2.37×10^−1^	0.355	
	Rel. foot mass	−5.41	**0.030**	
	Rel. foot length	1.42	**<0.001**	
	Sex (Male)	−1.30×10^−2^	0.097	
	Froude no.×body mass	−6.90×10^−4^	**0.003**	
	Froude no.×rel. foot length	−4.18×10^−1^	**0.018**	

Interaction effects between Froude number and anthropometric measure were only included in the model when significant. Marginal *R*^2^ represents the variance accounted for by the fixed effects of the linear mixed-effect model. Bold indicates significance.

### Stride frequency

In walking, individuals with greater relative foot–shoe mass exhibited lower stride frequencies (*P*=0.031).

In running, individuals with greater relative leg length exhibited higher stride frequencies (*P*=0.039). Additionally, with increasing Froude number (e.g. increasing speed), individuals with a greater relative foot–shoe mass increased their stride frequency less than others (*P*<0.001).

### Stride length

With increasing Froude number, heavier individuals and those with greater relative foot–shoe mass increased their stride length more than others in both walking and running (*P*≤0.049).

### Stance time

Heavier individuals (*P*<0.001) and those with greater relative foot length (*P*=0.033) exhibited longer stance times in walking. However, the effect of body mass on prolonged stance time diminished with increasing Froude number (*P*<0.001).

Similar to walking, heavier individuals (*P*<0.001) and those with a greater relative foot length (*P*<0.001) had longer stance times during running, but these effects decreased with Froude number (*P*≤0.025). Furthermore, on average females exhibited 7.2% longer stance times while running (*P*=0.006).

### Leg swing time

Individuals with a greater relative foot–shoe mass adopted longer leg swing times (*P*=0.006). Similarly, greater relative foot–shoe mass also increased leg swing time in running (*P*=0.007). In contrast, heavier individuals (*P*=0.010) and those with greater relative foot length (*P*=0.037) adopted shorter leg swing times. The effect of body mass on leg swing time decreased with Froude number (*P*<0.001).

### Duty factor

Greater relative foot–shoe mass was linked to lower duty factors in walking (*P*=0.030) and running (*P*=0.030), consistent with the observed longer swing time and unchanged stance time. Conversely, heavier individuals and those with greater relative foot length had higher duty factors in walking (*P*<0.001) and running (*P*≤0.001). However, the effect of body mass on duty factor diminished with Froude number in both gaits (*P*≤0.003), whereas the effect of relative foot length with increasing Froude number only reduced in running (*P*=0.018). Additionally, in walking, individuals with greater relative leg length reduced their duty factor more compared with others with increasing Froude number (*P*=0.024).

### Double support time

Heavier individuals (*P*<0.001) and those with greater relative foot length (*P*<0.001) adopted longer double support times. The effect of body mass on prolonged double support time reduced with Froude number (*P*<0.001). Individuals with greater relative leg length reduced their double support time more with increasing Froude number (*P*=0.011). Females exhibited 5.0% longer double support times compared with males (*P*=0.017).

## DISCUSSION

In this study, we investigated whether inter-individual anthropometric differences, such as body mass, relative foot–shoe mass, relative leg length, relative foot length and sex, could explain spatiotemporal gait variability beyond what is accounted for by Froude number. As expected, Froude number alone captured a large proportion of the variance in spatiotemporal parameters across individuals and speeds, exceeding 70% for all variables during walking and at least 47% for most spatiotemporal variables during running (Fig. 1). An exception was leg swing time, for which Froude number accounted for only 1% of the observed inter-individual variance. Incorporating anthropometric factors further improved model performance, capturing 77–93% of the spatiotemporal variance in walking and 16–94% of the spatiotemporal variance in running. Consistent with our first hypothesis, greater body mass was associated with longer stance times and higher duty factors, particularly at slower speeds, without affecting stride frequency. Interestingly, in walking, this prolonged stance time with body mass was primarily driven by increased double support time, as single leg support time (i.e. leg swing time of the contralateral leg) remained unaffected. Supporting our second hypothesis, greater relative foot–shoe mass reduced stride frequency and increased stride length, largely through longer leg swing times, and therefore also contributed to a lower duty factor in both walking and running. Another main finding was that individuals with greater relative foot length exhibited longer stance times and higher duty factors in both locomotion modes, again without altering stride frequency. In walking, this effect was mainly due to prolonged double support time, with single leg support time being unaffected. In running, longer feet relative to leg length also shortened leg swing time, but its influence on stance time and duty factor diminished with increasing speed. Sex-related effects were limited, although females had longer double support times in walking and longer stance times in running compared with males.

Under dynamic similarity, individuals moving at the same Froude number are expected to exhibit invariant dimensionless spatiotemporal characteristics. Although our models adjust for Froude number (note that we did not impose equal Froude numbers experimentally), the significant anthropometric effects indicate systematic deviations from dynamic similarity. These deviations are consistent with violations of geometric similarity (e.g. variable relative segment lengths and mass distributions) and with inertial effects of distal mass that disproportionately influence swing mechanics. Hence, even after controlling for Froude number, human anthropometric variability produces shifts in preferred spatiotemporal characteristics, rather than the invariance that dynamic similarity theory would suggest (Alexander and Jayes, 1983).

Our findings on anthropometric effects on spatiotemporal variability align with studies that experimentally altered segment masses or lengths. We observed that heavier individuals had longer stance times, higher duty factors and prolonged double-support times during walking, patterns similar to those reported when mass is added around the waist, which increases these parameters in walking (Browning et al., 2007; Griffin et al., 2003) and increases stance time and duty factor in running (Werkhausen et al., 2019) without affecting stride frequency. While we found that the influence of body mass on these spatiotemporal variables diminished with increasing speed, only Werkhausen et al. (2019) reported similar effects in running. Browning and Kram (2007) further showed that obese individuals (∼65% greater body mass) walking at 1.25 m s^−1^ exhibited ∼3.6% longer stance times, driven by ∼26.1% longer double support times and ∼5.1% higher duty factors, without differences in stride length or frequency as compared with healthy-weight individuals (Browning and Kram, 2007).

Our observation that greater relative foot–shoe mass reduced stride frequency by increasing leg swing time agrees with studies reporting decreased stride frequency and prolonged leg swing time when adding external mass to the foot, thereby directly increasing relative foot–shoe mass and the moment of inertia, in both walking (Browning et al., 2007) and running (Coifman et al., 2024b; Martin, 1985). The longer stance and double support times with greater relative foot length may relate to heel-to-toe roll of the foot, where larger relative foot lengths prolong stance and increase duty factor. Indeed, Adamczyk and Kuo (2013) demonstrated that increasing foot length by attaching rigid arc-shaped foot bottoms prolonged double support time (Adamczyk and Kuo, 2013). In running, not all runners adopt a heel-to-toe roll (e.g. rearfoot strike), yet rearfoot striking is the most common pattern (Hasegawa et al., 2007; Kasmer et al., 2013; Larson et al., 2011) and is associated with longer stance times compared with midfoot or forefoot striking (Di Michele and Merni, 2014; Hayes and Caplan, 2012; Mercer and Horsch, 2015). With increasing speed, runners tend to shift initial contact anteriorly toward midfoot or forefoot strike (Breine et al., 2014), reducing the functional foot length (e.g. the distance the center of pressure travels under the foot during stance). This transition might explain why the influence of relative foot length on stance time and duty factor diminishes at higher Froude numbers.

**Fig. 4.**
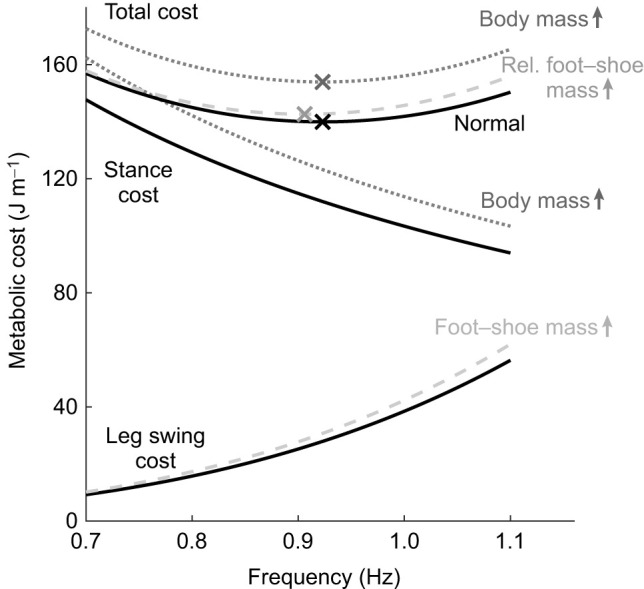
**Theoretical framework for how the metabolically optimal stride frequency for walking – and therefore presumably the preferred stride frequency – shifts with altered relative foot–shoe mass but not with increased body mass.** Leg swing cost (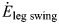
∼frequency^4^; Doke et al., 2005), stance cost (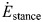
∼frequency^3^×length^4^; Donelan et al., 2002) and total metabolic cost as a function of stride frequency with an average body mass (black solid line), greater body mass (dark gray dotted line) and greater (relative) foot–shoe mass (light gray dashed line). Crosses indicate the metabolically optimal stride frequency. This theoretical model estimates a 1.8% decrease in optimal stride frequency with a 10% increase in relative foot–shoe mass, corresponding well with our experimentally observed decreased stride frequency in individuals with greater relative foot–shoe mass (e.g. −1.3% in walking and −1.1% to −2.4% in running; Tables S1 and S2).

Spatiotemporal gait variability might reflect individual strategies to minimize individual metabolic cost. A large body of previous work suggests that individuals adopt spatiotemporal gait parameters that minimize the metabolic cost of locomotion. The well-established U-shaped relationship between stride frequency and metabolic cost (Minetti et al., 1995; Umberger and Martin, 2007) likely arises from a trade-off between minimizing stance cost without excessively increasing leg swing cost (Fig. 4). The stance phase is metabolically expensive because of step-to-step transition work in walking (Donelan et al., 2002; Gottschall and Kram, 2005; Kuo et al., 2005) and body weight support and propulsion in running (Arellano and Kram, 2014; Chang and Kram, 1999; Teunissen et al., 2007). In walking, higher stride frequency reduces transition cost (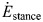
∼frequency^3^×length^4^) (Donelan et al., 2002), but leg-swing cost rises steeply at high frequencies (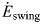
∼frequency^4^) (Doke et al., 2005), making short strides metabolically inefficient (Fig. 4). Greater relative foot–shoe mass, which increases leg moment of inertia, likely elevates swing cost without substantially affecting stance cost. Consequently, a lower stride frequency – enabled by prolonged leg swing time and thus shorter duty factor – may be metabolically optimal for these individuals (Fig. 4). In contrast, body mass did not significantly influence preferred stride frequency in either gait, yet heavier individuals adopted longer stance times and greater duty factors. In walking, this was primarily driven by prolonged double support. Because step-to-step transition cost reflects the energy required to redirect COM velocity between steps, body mass is a key determinant of this cost. As this redirection occurs mainly during double support, heavier individuals may extend this phase, allowing smoother transitions, reducing transition cost and consequently the metabolic cost of walking. This interpretation aligns with evidence that obese individuals exhibit longer double support times than healthy-weight individuals (Browning and Kram, 2007). In running, the ‘cost of force’ hypothesis posits that metabolic cost while running is proportional to body weight and inversely proportional to stance time (Kram and Taylor, 1990). Peak vertical ground reaction forces reach 2–4 times body weight over very short durations, requiring high rates of force development and energetically costly muscle activation. Increasing duty factor by prolonging stance reduces peak forces and distributes them over a longer period, lowering force-rate demands and associated energetic cost (Beck et al., 2020). This strategy may be particularly beneficial for heavier individuals, who must generate greater peak forces. Supporting this, Werkhausen et al. (2019) showed that externally added body mass did not increase triceps surae muscle activity because runners compensated by lengthening stance and increasing duty factor, thereby preserving or even reducing fascicle shortening velocity despite the added load.

Although Froude number and anthropometric characteristics accounted for most of the inter-individual spatiotemporal variability in walking (*R*^2^≥0.77), the model performed worse in explaining variability for running. While stride length was strongly associated with Froude number (*R*^2^=0.936), combining Froude number with anthropometric predictors only accounted for 16–74% of the variance in the other spatiotemporal variables (Fig. 1), suggesting that running gait is influenced by additional factors. As described earlier, one such factor is foot strike pattern, as non-rearfoot strike running is associated with shorter stance times (Di Michele and Merni, 2014; Hayes and Caplan, 2012; Mercer and Horsch, 2015). Other proposed influences include flexibility, strength and muscle fiber-type distribution. Dynamic stretching can reduce stride frequency by prolonging leg swing time (Caplan et al., 2009; Pappas et al., 2017), yet hamstring passive range of motion appears unrelated to spatiotemporal variables in adolescent runners (Garcia et al., 2022). Moreover, evidence contradicts the idea that strength or muscle fiber-type distribution strongly determines locomotion preferences as neither strength training (Albracht and Arampatzis, 2013; Bohm et al., 2021; Ferrauti et al., 2010; Roche-Seruendo et al., 2018; Trowell et al., 2020, 2022) nor muscle fiber-type distribution (Swinnen et al., 2024) affect spatiotemporal parameters. Running faster requires increases in both stride length and frequency, but increases in stride length contribute more at lower speeds, while stride frequency plays a larger role at higher speeds (Table 2). Individuals with lower running ability (e.g. lower aerobic speed) may begin increasing their stride frequency earlier, potentially adding to variability beyond anthropometric and Froude number effects. Overall, while anthropometric factors such as body mass, relative foot–shoe mass and foot length explain some variability, inter-individual differences in running appear highly multifactorial.

A methodological limitation of this study is that participants were assessed at fixed absolute speeds rather than at fixed Froude numbers. Dynamic similarity theory makes predictions for individuals moving at equal dimensionless speeds, and a strict scaling analysis therefore requires measurements at identical Froude numbers. Although our linear mixed–effects models appropriately adjust for Froude number and quantify anthropometric effects across a range of speeds, they do not constitute a formal test of dynamic similarity in the strict theoretical sense. However, complementary fixed–Froude number scaling analyses based on interpolation produced results that were highly consistent with the mixed–effects models (Figs S4 and S5). Additionally, this study focused on five easily measurable anthropometric characteristics, which may not fully capture an individual's anthropometry. Nevertheless, our findings suggest these characteristics represent key contributors to spatiotemporal variability. We collected spatiotemporal data on a treadmill, which facilitates controlled speeds and large numbers of strides being collected but may not perfectly replicate overground locomotion. However, spatiotemporal differences between treadmill and overground locomotion are minor and unlikely to be clinically relevant (Semaan et al., 2022; Van Hooren et al., 2020). Lastly, our sample consisted of regular runners and thus relatively fit participants, reflected in their BMI, with only seven individuals having a BMI over 25 and none over 27. Because we found that anthropometry influences walking more strongly than running, future studies focusing on walking should include participants who are classified as overweight or obese to capture greater variability. Such studies may reveal even stronger correlations. For example, the mean duty factor in our sample aligns with values reported for healthy-weight individuals (Browning and Kram, 2007) but is ∼2.8 percentage points (3.4 times our standard deviation) lower than that observed in obese individuals walking at 1.25 m s^−1^.

In conclusion, our findings indicate that individual differences in anthropometry help explain variability in spatiotemporal gait patterns in walking and, to a lesser extent, in running. We suggest that these adaptations reflect individual strategies to minimize the metabolic cost of locomotion but this should be confirmed in future work.

## Supplementary Material

10.1242/jexbio.252161_sup1 Supplementary information

Dataset 1.
