# Supplementary material for: Body anthropometry affects spatiotemporal preferences in walking and running
Source: J Exp Biol. 2026 May 19;229(10):jeb252161. doi: 10.1242/jeb.252161 (PMC13245910; doi:10.1242/jeb.252161)
Supplement: Supplementary information [file jexbio-229-252161-s1.pdf]

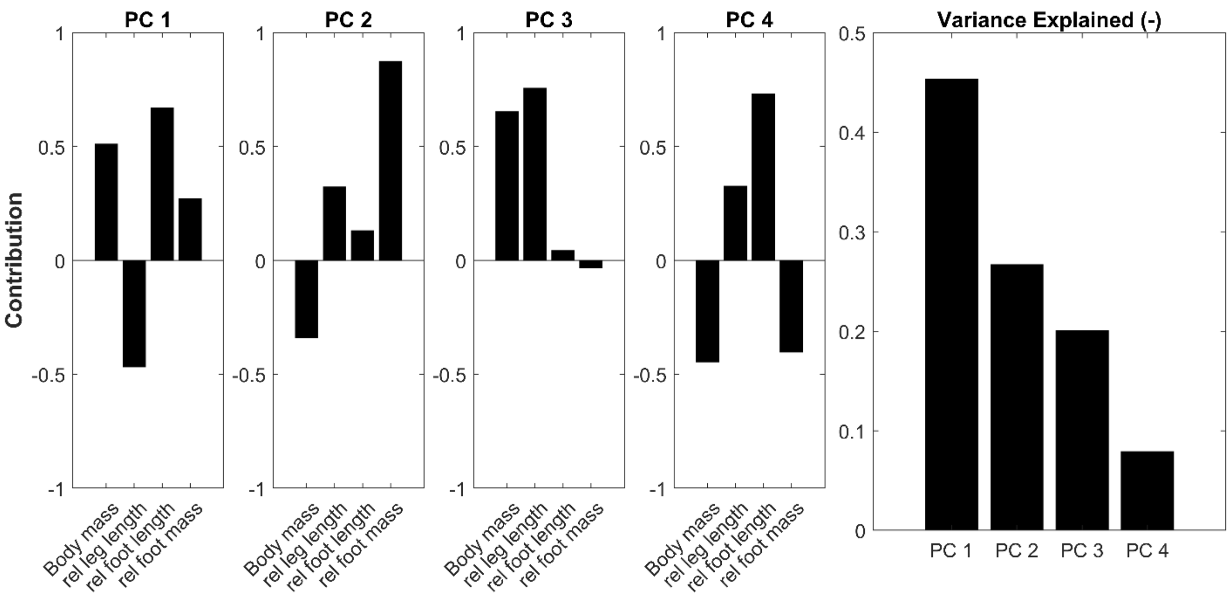

Fig. S1. Principal component analysis of the collinearity between anthropometric characteristics.

Journal of Experimental Biology • Supplementary information

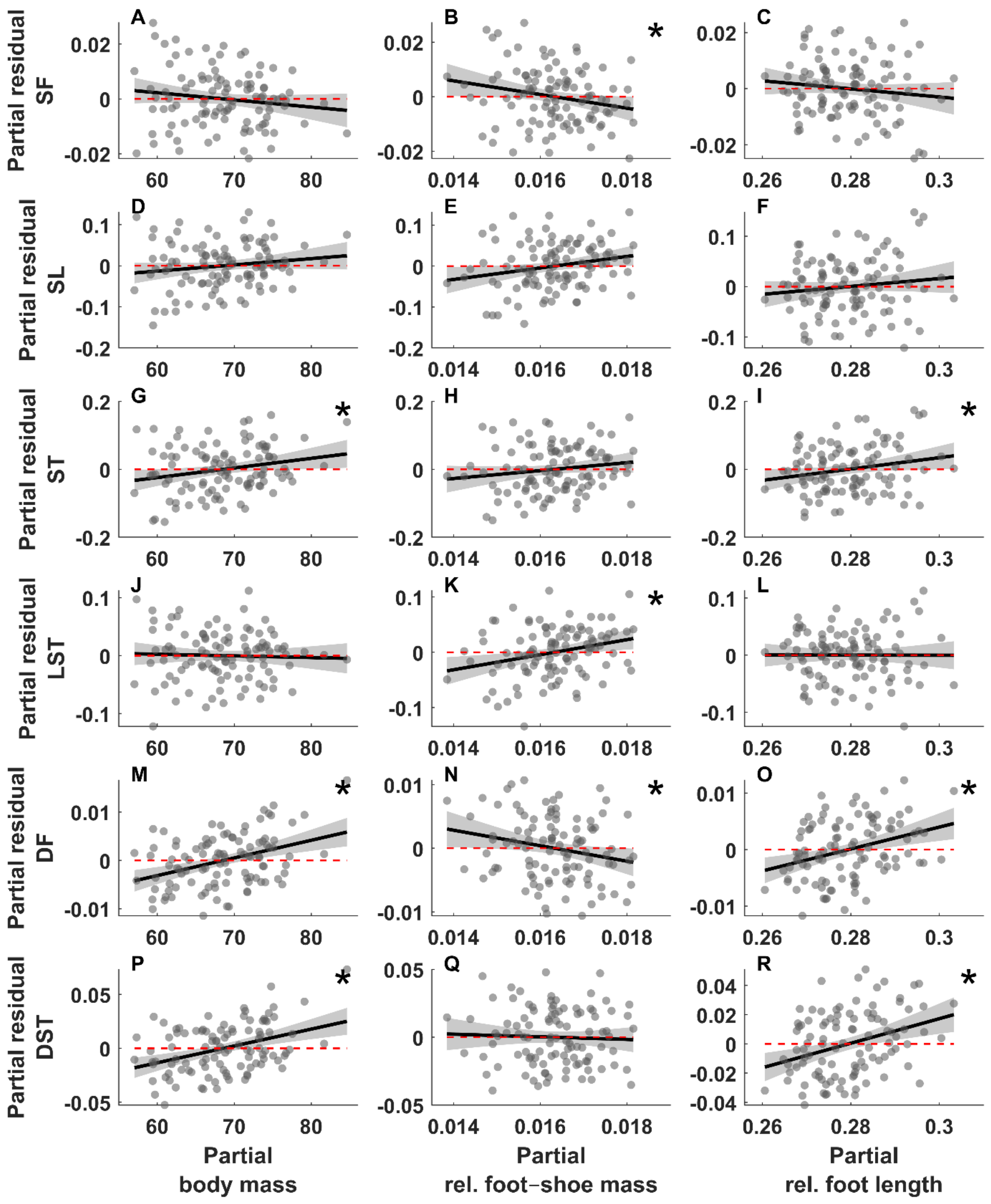

Fig. S2. Partial (added-variable) regression plots showing the unique effects of anthropometric predictors on dimensionless spatiotemporal gait variables during walking. Partial residuals represent variation in each gait variable after removing the fixed effect of Froude number and adjusting for the other anthropometric predictors and sex. Partial predictors represent variation in each anthropometric measure after adjusting for the remaining predictors and sex. Black lines show fitted partial slopes with 95% confidence intervals; grey points represent participants. Red dashed lines indicate the null slope (0) expected under dynamic similarity for dimensionless variables at equal Froude number. * indicates significant main effects of anthropometric predictors in the LMEM. SF = stride frequency; SL = stride length; ST = stance time; LST = leg swing time; DF = duty factor; DST = double support time. For clarity, relative leg length is not shown because it did not exhibit a significant main effect in the LMEM.

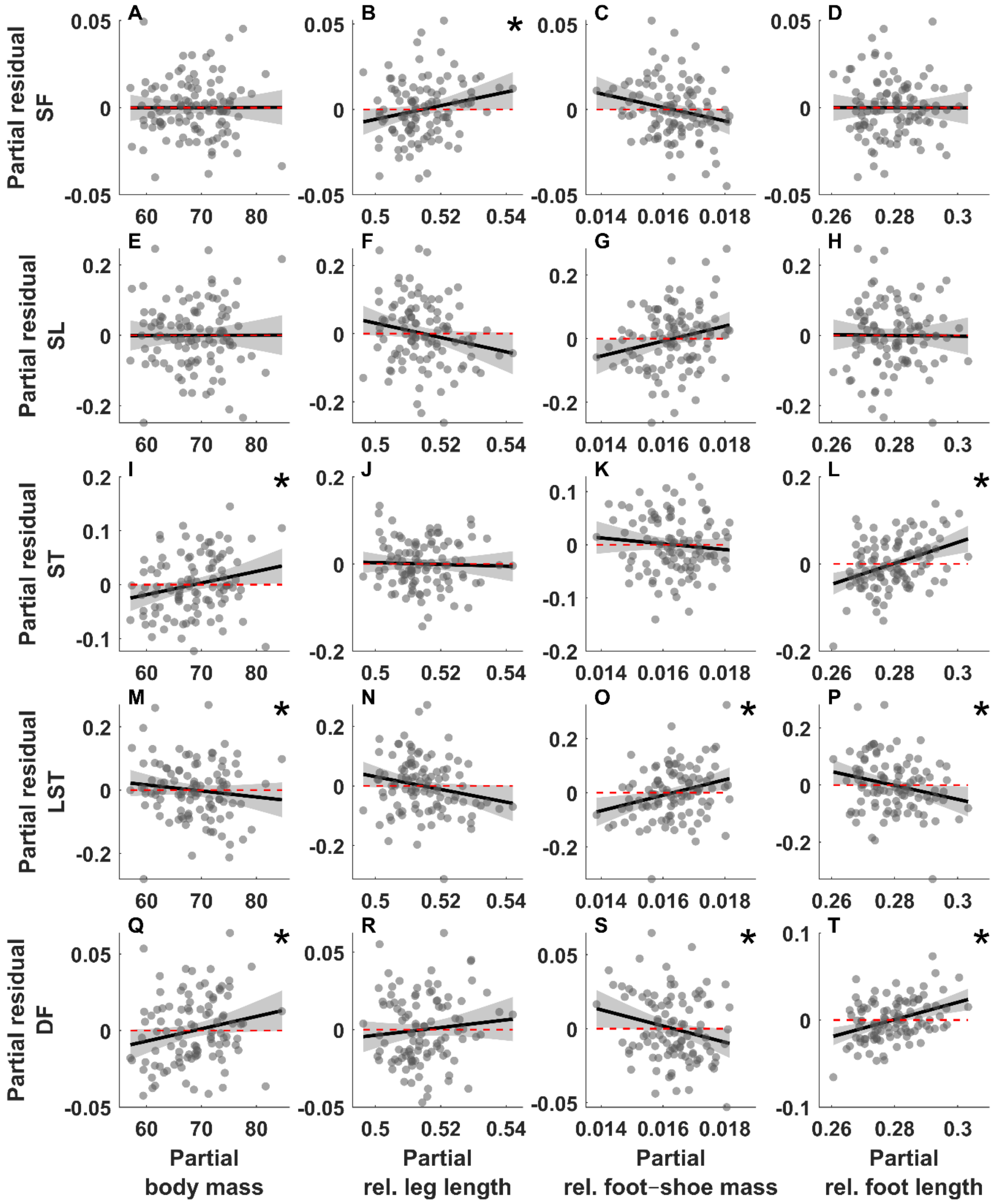

Fig. S3. Partial (added-variable) regression plots showing the unique effects of anthropometric predictors on dimensionless spatiotemporal gait variables during running. Partial residuals represent variation in each gait variable after removing the fixed effect of Froude number and adjusting for the other anthropometric predictors and sex. Partial predictors represent variation in each anthropometric measure after adjusting for the remaining predictors and sex. Black lines show fitted partial slopes with 95% confidence intervals; grey points represent participants. Red dashed lines indicate the null slope (0) expected under dynamic similarity for dimensionless variables at equal Froude number. * indicates significant main effects of anthropometric predictors in the LMEM. SF = stride frequency; SL = stride length; ST = stance time; LST = leg swing time; DF = duty factor.

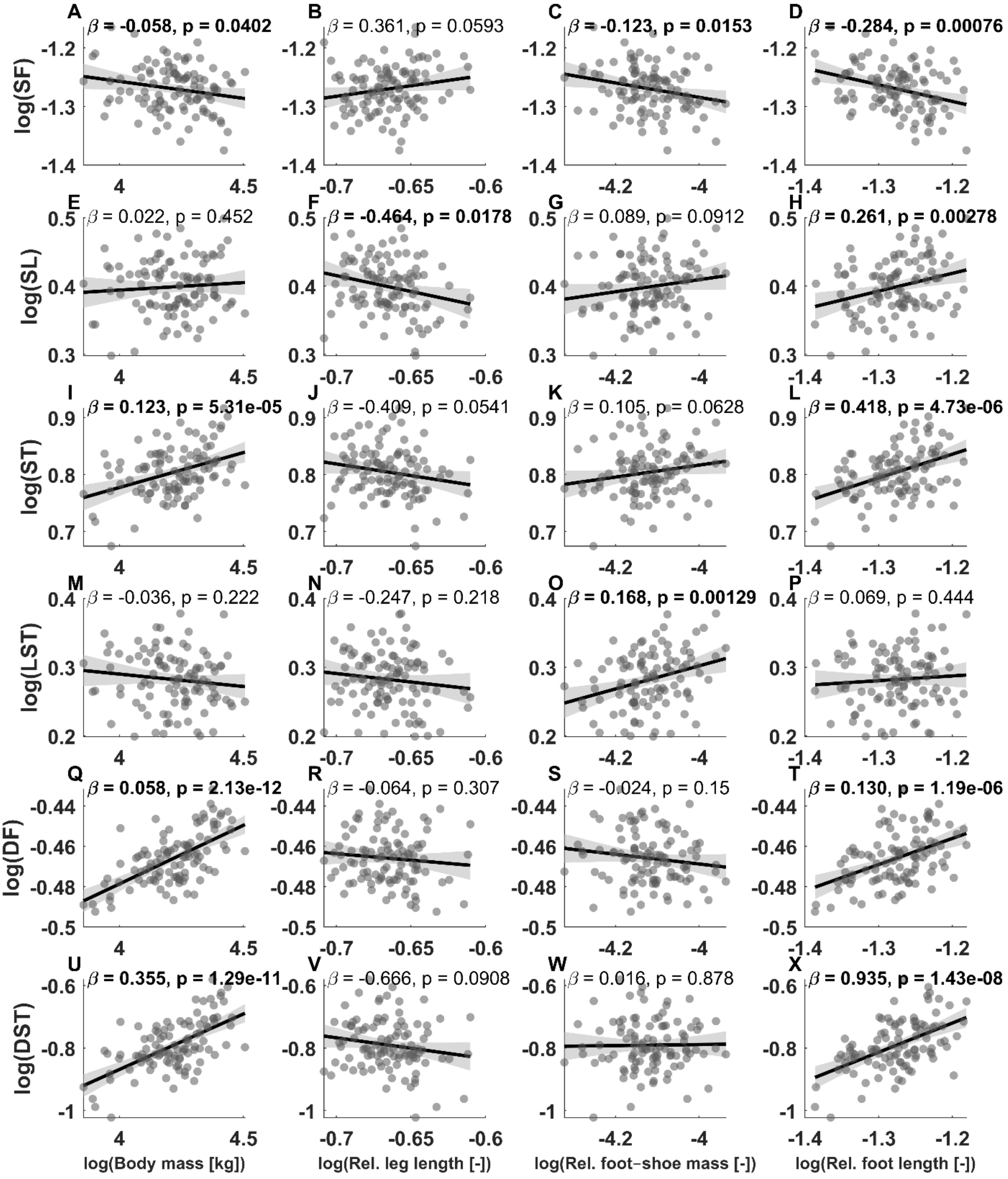

Fig. S4. Scaling analysis of dimensionless spatiotemporal variables during walking at a fixed Froude number (Fr = 0.175). Each points represents an individual participant; black lines show least-squares regression fits in log-log space; shaded regions indicate 95% confidence intervals of the mean predicted value. Under dynamic similarity, dimensionless gait variables should be size-invariant at equal Froude number, implying a scaling exponent of $\beta \approx 0$. Estimated slopes (β) that differ significantly from zero ($p < 0.05$) are shown in bold. SF = stride frequency; SL = stride length; ST = stance time; LST = leg swing time; DF = duty factor; DS = double support time.

Journal of Experimental Biology • Supplementary information

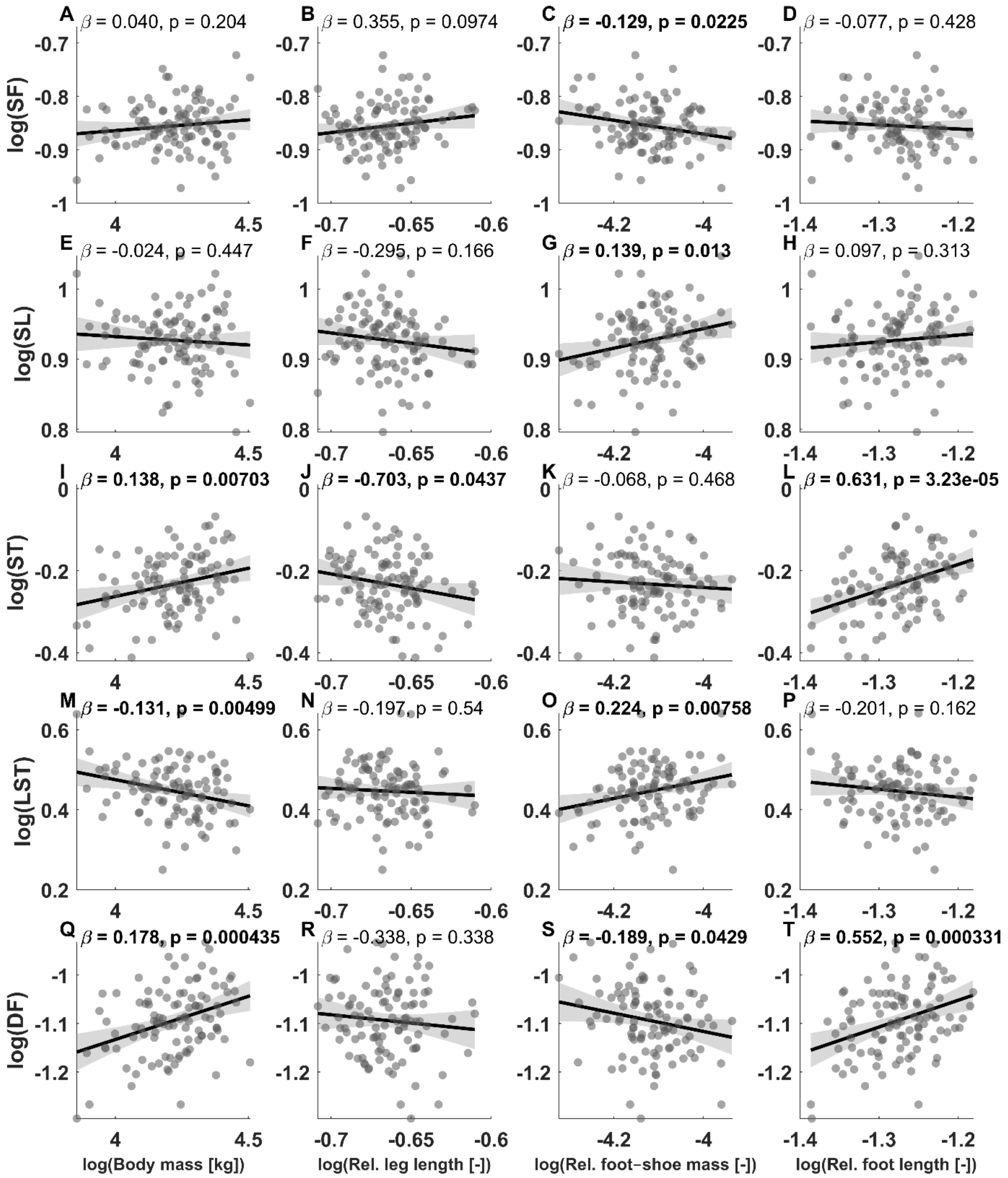

Fig. S5. Scaling analysis of dimensionless spatiotemporal variables during running at a fixed Froude number (Fr = 1.25). Each points represents an individual participant; black lines show least-squares regression fits in log-log space; shaded regions indicate 95% confidence intervals of the mean predicted value. Under dynamic similarity, dimensionless gait variables should be size-invariant at equal Froude number, implying a scaling exponent of $\beta \approx 0$. Estimated slopes (β) that differ significantly from zero ($p < 0.05$) are shown in bold. SF = stride frequency; SL = stride length; ST = stance time; LST = leg swing time; DF = duty factor.

Journal of Experimental Biology • Supplementary information

Table S1. The predicted effect of an isolated 10% increase in each anthropometric characteristic on the spatiotemporal gait preference when walking at a Froude number of 0.175 (~1.25 m/s) and 0.45 (~2 m/s). If an interaction effect between Froude number and the anthropometric characteristic was observed, separate values were computed at each Froude number. If no interaction effect was observed, the mean of both Froude numbers is reported. Coefficients which were not significantly different from zero, were not considered and presented as "no effect". Note that the variability of each of the anthropometric characteristics is different, emphasized by how many times the standard deviation in our sample corresponds with a 10% increase in the anthropometric characteristics (between brackets).

WALKING	Stride frequency		Step length		Stance time		Leg swing time		Duty Factor		Double support time	
Froude number	0.175 ~1.25m/s	0.45 ~2 m/s	0.175 ~1.25m/s	0.45 ~2 m/s	0.175 ~1.25m/s	0.45 ~2 m/s	0.175 ~1.25m/s	0.45 ~2 m/s	0.175 ~1.25m/s	0.45 ~2 m/s	0.175 ~1.25m/s	0.45 ~2 m/s
+10% Body mass (0.7*SD)	No effect		+0.3%	+0.9%	+1.3%	+0.5%	No effect		+0.6%	+0.2%	+3.8%	+1.4%
+10% Relative leg length (4.9*SD)	No effect		No effect		No effect		No effect		+0.6%	-0.4%	+0.4%	-7.9%
+10% Relative foot-shoe mass (1.3*SD)	-1.3%		+1.1%	+1.5%	No effect		+1.8%		-0.3%		No effect	
+10% Relative foot length (2.2*SD)	No effect		No effect		+2.4%		No effect		+0.9%		+6.7%	

Table S2. The predicted effect of an isolated 10% increase in each anthropometric characteristic on the spatiotemporal gait preference when running at a Froude number of 0.7 (~2.5 m/s) and 1.95 (~4.17 m/s). If an interaction effect between Froude number and the anthropometric characteristic was observed, separate values were computed at each Froude number. If no interaction effect was observed, the mean of both Froude numbers is reported. Coefficients which were not significantly different from zero, were not considered and presented as "no effect". Note that the variability of each of the anthropometric characteristics is different, emphasized by how many times the standard deviation in our sample corresponds with a 10% increase in the anthropometric characteristics (between brackets).

RUNNING	Stride frequency		Step length		Stance time		Leg swing time		Duty Factor	
Froude number	0.7 ~2.5 m/s	1.95 ~4.17 m/s	0.7 ~2.5 m/s	1.95 ~4.17 m/s	0.7 ~2.5 m/s	1.95 ~4.17 m/s	0.7 ~2.5 m/s	1.95 ~4.17 m/s	0.7 ~2.5 m/s	1.95 ~4.17 m/s
+10% Body mass (0.7*SD)	No effect		-0.3%	+0.4%	+2.2%	+0.6%	-1.3%	+0.2%	+2.0%	+0.5%
+10% Relative leg length (4.9*SD)	+4.8%		No effect		No effect		-7.0%		No effect	
+10% Relative foot-shoe mass (1.3*SD)	-1.1%	-2.4%	+1.0%	+2.6%	No effect		+3.0%		-2.7%	
+10% Relative foot length (2.2*SD)	No effect		No effect		+8.8%	+7.0%	-4.4%		+8.8%	+6.2%

Dataset 1.

Available for download at
https://journals.biologists.com/jeb/article-lookup/doi/10.1242/jeb.252161#supplementary-data

Journal of Experimental Biology • Supplementary information
